# Please Excuse Us

**Published:** 1871-02

**Authors:** 


### PLEASE EXCUSE US.

Our friend, Dr. ———, will please excuse us for declining to publish his communication. While his statements are true, no doubt, we do not desire, and cannot make our Journal the medium for the ventillation of private wrongs, or individual grievances. Our aims are higher—nobler than this. We will at all times be happy to lend our pages to the vindication of principles, or to the discussion of the true ethics of the profession; and if, in the advocacy of these principles and truths, our remarks, or the arguments of our friends, *based upon principles* underlying the ethics which should govern all, should seem to fasten upon any, then the fault will not be ours, or that of our friends.

If Dr. will make an argument, stripped of personalities, showing what should or should not be tolerated by the profession, we will gladly give him a place in our journal, with or without his real signature—as he may desire.

We can appreciate the reasons which often make it desirable on the part of correspondents to withhold their names from the public; and while we would greatly prefer always to give the true signature of the author, yet we will waive this preference, when the articles, within themselves, contain valuable matter of interest for the good of the profession.

